# Observations on the Deranged Manifestations of the Mind, or Insanity

**Published:** 1818-06

**Authors:** 


### *Observations on the Deranged Manifestations of the Mind, or Insanity.* By J. G. Spuzrheim, M. D. &c.

( Continued from page 420. )

We have already put our Readers in possession of the plan of this work, and we now come to the principal division of it—that which treats exclusively of Insanity. The Second Chapter of Part II. contains the seven following Sections or subdivisions. 1st. The name and definition; 2d. The symptoms; 3d. The division of Insanity;

4th. Its causes; 5th. Its forms; 6th. Its prognosis; and, 7th. The treatment, moral and medical.

The Author's definition of mental alienation is as follows.

"Insanity is an aberration of any sensation or intellectual power from the healthy state, without being able to distinguish the diseased state; and the aberration of any feeling from the state of health, without being able to distinguish it, or without the influence of the will on the actions of the feeling. In other words, the incapacity of distinguishing the diseased functions of the mind, and the irresistibility of our actions, constitute Insanity." P. 71.

This definition is by no means unexceptionable; but it would be a waste of time to start captious objections on such a matter. In truth, we think, that a definition of Insanity, like that of a wound, or of any thing equally obvious, is too often merely a round-about attempt to explain, in measured and pedantic language, a thing sufficiently understood. Hence, such definitions are thoroughly superfluous, and tend to darken what is clear, or to complicate that which is simple. Notwithstanding the supposed perfection of the one just given, the Author, in the remainder of the Section, speaks of the great difficulties we meet with in examining this disorder, as the object of Juridical Medicine, in cases where it is partial or intermittent.

2. *The symptoms.* The great variety of the symptoms of this disorder, which are everlastingly varied, and are even opposite in different cases, Dr. S. contends, can only be accounted for by his peculiar metaphysical views of the mind; and this leads us to state briefly what these views fundamentally are. He considers that the mind, or, as Dr. Hutcheson of Glasgow defines it, the "animus qui cogitat, aut cogitare potest," is not an essence whose *modes* (as Philosophers call them) produce the various phænomena of mental operations, but that it consists rather of an aggregation of various primitive powers, which may be manifested or not, independently of each other, though in the state of health, and often even in disease, they exercise a most important mutual influence This congeries of powers, he conceives, may be aptly divided into Faculties and Feelings; the former comprehending the intellectual and higher moral and reflective powers, which in the language of Religion are called "the reasonable Soul;" and the latter embracing the whole compass of human passions, instincts, and affections. He contends, from observation, that each primitive power, and primitive feeling, has a separate portion of the cerebral organization for the medium of its manifestations, and that one or more of these portions of the nervous mass of the brain may be excited by sanguineous impulse, or deranged in its functions, mediately or immediately, by various causes; and hence may give rise to alienation of that mental power or feeling whose organ it is, while the other cerebral parts and mental operations remain undisturbed.

We do not choose to offer any remarks for or against this Theory, because such a proceeding would lead us into a labyrinth of speculation, no way edifying either to ourselves or our readers; and because we know that the theory of the human mind has mocked the research of Philosophers in all ages, from Aristotle downwards. We shall simply transcribe the following strong passage, by which Dr. S. enforces his general considerations.

"Those who consider the manifestations of the mind as independent of the body, or even our common knowledge of the influence of the body on the mind, will not explain an infinite number of insane appearances. How will the former account for the cases of ideots from birth? Have those individuals no mind? How can they understand why, in others, the independent mind, endowed with reflection and will, is mischievous beyond measure? Or, that others think themselves the vilest of the vile, without any previous fault? Where is the mind in those who sit whole days with their eyes immoveably fixed on one object, and seem wholly absorbed in their own contemplation, and refuse all kind of nourishment? How will they account, by the mind, that some are given up to the most brutal instincts, and others disposed to devotional melancholy, and continually engaged in penetrating the hidden mysteries, combating the various opinions of different sects, and in propagating their own? The doctrine of temperaments is not more fit to explain such disorders of the mind. There is no temperament peculiar to idiotism, mania, or melancholia. Many melancholic patients have the external characters of a sanguine temperament, such as a fair complexion, a fine skin, clear coloured hair, and blue eyes; or the signs of a bilious temperament, as a lean dry frame, sallow skin, a complexion of a brownish yellow colour, dark black stiff hair, and dark sunk eyes. Finally, the diseases of the abdominal viscera are not sufficient to explain insanity. In many idiots the viscera are in perfect health; and in many cases the viscera are greatly diseased, but there is not the slightest derangement of the mind. The physiology and pathology of the brain alone can explain the manifestations of the mind in the state of health or disease." P. 173, 174.

Upon the whole he concludes, that as in Insanity some faculties are improved, and even new ones evolved which the person did not possess before, while others are impaired or suppressed; as some patients are gay, lively, and loquacious, while others are gloomy, taciturn, and morose; some bold, others timid:—as some conceive themselves to be popes, emperors, or divinities, while others are transformed into felons, thieves, and ferocious murderers, on the watch to steal or break every thing in their way, and to imbrue their hands in human blood,—the inference is legitimate,

"That all these phænomena can be explained only by the plurality of independent faculties and respective organs, and their mutual influence." P. 89.

As to the division of Insanity, the author very freely criticises that adopted by Dr. Arnold, as also Mr. Hill's, and the more ancient, though least philosophical one, by which this disorder is made to consist of mania and melancholia; these, it is obvious, are merely sorts of alienations. Dr. Spurzheim's own division is a much more just and luminous one; it is this: 1st. Idiotism, partial or general. 2d. Fatuity, partial or general. 3d. Irresistibility, i.e. where the will has no influence upon the actions. 4th. Alienation, where the faculties are deranged in their quality, and the intellect is incapable of distinguishing the derangement.

The 4th Section, on the Causes of Insanity, is, in our opinion, by far the most valuable and original one in the volume; it is valuable not only in a theoretical but a practical sense, inasmuch as an accurate knowledge of the causes of insanity, more perhaps than of any other disease, is not only of great interest as a scientific attainment, but also sheds much light upon its treatment, whether moral or medical, and is often even indispensibly necessary to that right treatment. The author first maintains and illustrates the two propositions, that "the proximate cause of insanity is corporeal;" and that "this proximate cause is in the brain;" and then expatiates, with much compass of erudition, and originality of illustration, on the nature of these causes, whether proximate or remote, whether idiopathic, dynamic, or sympathic. We regret that our limits will only permit us to mention the heads of the subjects treated of; yet we hope that their importance will induce the reader to study them at large in the work itself. After premising that

"The mind in this life is confined to the body of which it makes use; that the powers of the mind want instruments for their manifestations, and that these manifestations are dependant on the instruments; cannot appear without them; and are modified, diminished, increased, or deranged, according to the condition of the instruments or organs."

He infers, that the proximate cause of insanity is corporeal from the following six considerations: 1st. The disease is connate and hereditary. 2d. It is influenced by age, and by the degree of evolution of the cerebral mass. 3d. It is produced by causes which evidently injure the body alone. 4th. It depends on season and weather, (though not on the influence of the moon or other planets, as ancient prejudice has taught). 5th. It is periodical, and has exacerbations, remissions, and intermissions like other diseases confessedly corporeal; and, 6th, It is often accompanied, or alternates with other corporeal diseases, such as irregular gout, rheumatismus vagus, worms, suppression of the catamenia, hæmorrhoides, lochia, old sores, or drains, &c.

To the proposition tha "the proximate cause of insanity is in the brain," it has been objected that there is not always a perceptible change of organization after death; and Dr. S. thus replies to it.

"The brain is an organic part, and we must not expect to find more in it than any other part of the body. Nay, as its organization is the most delicate of the whole frame, organic changes may occur which are imperceptible on dissection;* since this is also the case in other parts, which may be affected by various diseases, without offering the least morbid appearance after death. The stomach, for instance, and intestines have often suffered for a long time, and the most skilful anatomists cannot detect any thing different in their structure after death. On the other hand, neither vomiting nor want of appetite have [*has*] taken place in persons in whose stomach mortification was found after death. Abscesses have been observed in the livers of persons who have died without any one of the common symptoms of hepatitis." P. 116.

We select the following passage as a specimen of Dr. S's manner of considering the subject, and as exemplifying the way in which he applies the peculiar language

---

* The very same opinion is advanced by Dr. Armstrong in his inimitable work on Typhus. In persons who die of apoplexy, particularly when the fit has been brought on by mental causes, deviations from the healthy structure frequently cannot be detected. Many of Dr. Cheyne's cases prove this. In truth, our knowledge of the brain is quite in its infancy. Rev.

of his organological theory to the phænomena of mental alienations.

" The deranged functions of the brain and the morbid changes of the organization ought never to be separated with respect to pathology, any more than disturbed respiration and diseased lungs. The appearances of insanity are merely symptoms of the deranged functions of the organs of the mind. The organ of the feeling of *self-esteem* being deranged, must naturally produce symptoms different from the disorders of the propensity to *destroy*, or to *conceal*, or of *cautiousness*, or *benevolence*, &c.; hence, there are as many sorts of symptoms as primitive faculties of the mind, and their combinations. In this manner alone we can understand why melancholia and mania are often the same disease, may interchange with each other, and why the same treatment may be successful in both, and why in other cases they are different? In our physiological language I would say, in melancholia the organ of *cautiousness* suffers more, and in mania that of *combativeness* or *destructiveness*, or both: now in these cases, the morbific cause may be the same, or different. Moreover, as the same disease — gout for instance, or rheumatism, may affect different parts of the body, so the same morbific cause may attack successively different parts of the brain; and in this way alone can we explain why the same insane person, in one fit may be pious and say prayers; in another, may curse, and endeavour to kick, bite, and destroy every thing within his reach; may in one fit weep, in another laugh. Do we not see that the cause of hysteria now affects the lungs, then the stomach, the head, the teeth, the ears, eyes, and the different cerebral parts," &c. P. 118.

If there is any truth in any part of the views of Drs. Gall and Spurzheim more than in another, it is in that where they allege the uniform smallness of the brain in cases of congenital idiotism; and for our parts, despite of the fastidious fear of M. Pinel against too hasty conclusions on this point, we are ready to admit that there is a positive connection, in these cases, between idiotism and the defect of organization. Dr. S. has treated this part of the subject with praise-worthy copiousness.

That Insanity is frequent in England, more than in other countries, will scarcely admit of a doubt. Our author attributes this lamented circumstance to the greater energy and agitation of the mental faculties produced by the spirit of liberty, by the clashing of weighty interests, and the constant opposition of opinion, whether political or religious. Ambition also, and the discouragement of marriage, in consequence of expensive habits, sudden vicissitudes of fortune in a commercial community, the too great indulgence of the people in spirituous liquors, and a manner of living "not conformable to dietetic principles," together with the moisture and gloom of the climate, he conceives, rank amongst the chief moral and physical causes.

The 5th Section is on the forms of Insanity, and introduces to the reader some interesting histories of suicide. This, Dr. S. contends, is a corporeal disease, and is endemical in some countries, and in some districts of particular countries. He asserts, that, "like other forms of insanity, the inclination to self-destruction is often hereditary." P. 186.

We shall make no apology for the length of the following extract on the curious subject of *Periodicity*,—a subject which, so far as we know, has never been before hinted at by writers ancient or modern.

"The functions of the nervous system are exhausted, and its powers require rest to be repaired. They are excited and even deranged, by various stimuli, as light, caloric, galvanic fluid, and especially by blood. But the greater irritability of the nervous system at certain periods is unexplained. The fact that at certain times the irritability of the nervous system (nerves and brain) is greater than at others, is indubitable, and, as it seems, dependent on determinate laws, and on other phænomena of Nature which are not yet ascertained. These periods of irritability are of the highest importance in the state of health and disease, with respect both to automatic and animal life.

"It seems to be a great law of Nature, that all phænomena happen with a certain periodicity. Plants at two periods, in the spring, and about the end of July and the beginning of August, grow particularly in extent; at the other time of the summer and autumn the young shoots become solid, and the plants perform other functions, especially those of fructification. Animals are born and increase according to periods. The climacteric years are known, and then the body increases more than at other times. Moreover, it is a fact, that the different parts of the body, such as teeth, cerebral parts, sexual parts, are developed at different periods. During the whole life the change of matter of our body is greater at certain times than at others; the alvine evacuations are more abundant, the urine turbid, the exhalation of the skin and lungs more considerable; in short, the function of every part more or less active, at different periods. In animal life, it is the same; sleep is necessary, and the instinct of animals, all feelings of man, even the intellectual faculties, are more energetic at one time than at another.

"Many diseases require a certain lapse of time before the natural state can be re-established, and they are subject to certain periodicity. A philosophical treatise on the periodicity of the phænomena of Nature in general, and of man in particular, in his state of health and disease, would, at the same time, be very interesting for anthropology, and very useful for practical medicine. There is one sort of periods which I call the periods of irritability, which have an influence on man in general, but particularly on the manifestations of the mind.

"Dr. Gall first made the observation, that at certain periods, more women menstruate than at others, and that in a lunar month there are two such periods." P. 191, 192.

The author then dilates at considerable length on the subject of menstruation, and asserts that the knowledge of this periodicity may become useful in many practical cases, as "it is a fact that pregnant women are delivered at the tenth period of menstruation," and therefore that the accoucheur may be on his guard accordingly. But we confess that we have very strong doubts of the accuracy of this fact; yet it is worth being kept in view. Dr. Spurzheim resumes thus,

"These periods when women menstruate, have an influence on the whole of mankind, on the state of health and disease; they affect men and women, at the same time, over Europe. Almost every one feels from time to time, during a few days, a greater irritability; he is easily displeased with any impressions of the senses; his mind is not disposed to any application, and is easily fatigued; his thoughts are not consecutive; he may be offended by things which, at other times, would be indifferent; he is morose, and more inclined to quarrel or to dispute; his appetite is lessened, and all his excretions are more copious. This state comes and goes away, without our being able to account for it.

"These periods are extremely important in medicine; all chronic diseases have, at these times, exacerbations; those who suffer by piles are more tormented; many morbific causes, which are permanent, produce greater derangement. They have also their influence on nervous complaints, and all sorts of periodical fits, on visionaries, and on all madmen. They explain why suicide is more frequent at one time than at another; why sometimes their melancholy seems to be cured, but returns; why such individuals, being saved or prevented destroying themselves, after a few days, are glad to be alive; why, yet a short time after, they make new attempts to finish their existence; and why they repeat them three, four, five, and more times, till at last they succeed. The cause of this general influence is unknown." P. 194, 195.

With regard to the prognosis of Insanity, Dr. Spurzheim agrees with the opinion of the late very eminent Dr. Willis, that the disease is generally susceptible of cure when it has been of short standing, and *vice versa.*

Owing to the great length of our preceding observations and extracts, we are reluctantly compelled to make short work of the last and highly valuable section on the moral and medical treatment. The author's directions for the moral treatment of the insane are original, rational, and satisfactory, and must stamp a high value on the work, even had it contained nothing more. The principle which he lays down is, in fact, that there can be no general principle at all, but that the treatment of every case must be modified by the individual particulars, and the train of mental alienation.

The manifestations of the mind in Insanity are just as much varied as in health; consequently, the treatment must be varied according to the symptoms, taking care to withhold all stimuli from that faculty which is deranged, or that feeling which is unduly active.

" A sore or inflamed part of the body is not to be rubbed, an inflamed muscle is not to be moved, and an inflamed eye is not to be exposed to strong light. In the same way, any feeling, being already too active or alienated, ought not to be put into action."—— — " How injudicious is it, therefore, to give books to persons insane from religion, or to let them hear sermons, which nourish their disorders; or to keep, with melancholics, a conversation on the subject of their despondency! &c." P. 245, 246.

He subjoins a description and plate of a mad-house, with excellent directions for the improvement of the interior œconomy and management of such establishments. He humanely recommends the mildest coercion by the strait waistcoat, where coercion is absolutely necessary; and justly reprobates the barbarity of treating disease worse than crime; of loading the innocent insane with chains and stripes, while the most nefarious felons are allowed a greater share of moral and physical comforts!

In the medical treatment, the same general directions hold, namely, that there can be no uniform system of cure applicable to all cases. The author reprobates the routine, in terms of strong but just indignation, by which vomiting, bleeding, purging, and sousing with cold water, &c, &c. have been indiscriminately employed in almost all cases. With a reference to medical treatment, and how it should be modified according to the individual merits of any individual case, the author divides Insanity into the *hypersthenic,* the *asthenic,* and the *nervous,* each requiring a varied and even opposite plan of cure.

Although we, in a great measure, subscribe to the correctness of this division, we are nevertheless persuaded, that the *hypersthenic* is by far the most frequent form of this malady; and we conceive that modern pathology is deeply indebted to Drs. Parry and Armstrong, for having pointed out the extreme frequency of sanguineous impulse on the organization of the brain as a cause of maniacal and other analogous disorders.

The work seems to have been written and produced with too much haste; a circumstance highly blameable in the author, and for which we can scarcely pardon him. He would have done well, also, to have put his MS. into the hands of some English friend for revision, for the style manifests every where an unacquaintedness with the *tournures* and minuter idioms of our language. Yet these are comparatively petty faults, and the work is a very valuable one in spite of them. It well deserves a place in the library of the medical reader, hard by the meritorious volumes of Haslam, Cox, Rush, Pinel, Halloran, &c.; and we have no hesitation in saying, that he who undertakes the care and treatment of mental derangements, without having read and digested the important contents of this publication, will be greatly deficient in his duty.

The author deserves especial praise for the earnestness with which he has enforced the proposition that the brain is the seat of Insanity; and therefore, that it is a corporeal disease. In this, to be sure, he is not absolutely original, for all modern physiologists (with the exception of "Sir Oracle," in a certain celebrated Northern Review) are of opinion that the brain is the instrument of the functions of the mind both in health and disease; still, we assert, Dr. Spurzheim has the merit of having illustrated and enforced it more fully than any author heretofore. Indeed he has, perhaps, in this respect, a little overshot the mark; for he lays such stress on organization, that more than once, while perusing his volume, we conceived he was treading upon the very brink of materialism.

After this favourable specimen of his powers, it must be granted that Dr. Spurzheim has rebutted the busy slanderer's sneer, brushed off the swarm of punsters and small poets that lately buzzed around him, and finally put down his persecutors under his feet.

